# Optic Disc Elevation Secondary to Cerebral Malaria Resolves Completely With Mannitol Administration and Corresponds to Clinical Improvement

**DOI:** 10.7759/cureus.40639

**Published:** 2023-06-19

**Authors:** Matthew Douglas-Vail, James Fah, Alexander Cotran-Lenrow, Robert C Pintwala

**Affiliations:** 1 Department of Emergency Medicine, The University of British Columbia, Vancouver, CAN; 2 Emergency Medicine, Partners In Health Liberia, Harper, LBR; 3 Emergency Medicine, J.J. Dosen Hospital, Ministry of Health, Harper, LBR; 4 Microbiology, Partners In Health Liberia, Harper, LBR; 5 Department of Ophthalmology and Vision Science, The University of British Columbia, Vancouver, CAN

**Keywords:** emergency medicine, tropical infection, optic disc elevation, cerebral malaria, pocus

## Abstract

This case report describes a 14-year-old boy presenting with cerebral malaria in a resource-limited setting. The patient exhibited optic disc elevation, indicating increased intracranial pressure (ICP). Due to the unavailability of advanced neuroimaging, point-of-care ultrasound (POCUS) was employed to assess the optic disc. After administration of a weight-based dose of mannitol, optic disc elevation resolved completely, accompanied by clinical improvement. This case highlights the potential of POCUS as a valuable tool for the assessment and management of cerebral malaria in resource-limited settings.

## Introduction

Cerebral malaria (CM) is a severe manifestation of malaria infection characterized by neurological complications including altered mental status and coma. One of the potential complications associated with cerebral malaria is increased intracranial pressure (ICP), which can contribute to the severity and mortality of the disease. While the pathophysiology underlying increased ICP in cerebral malaria remains poorly understood, it has been identified as a significant prognostic factor [1]. Raised ICP occurs when there is an abnormal increase in pressure within the cranial vault, potentially leading to compression of vital structures and impaired cerebral perfusion. In the presence of raised ICP from the subarachnoid space along the optic nerve, the optic nerve sheath (ONS) expands [2]. In the context of cerebral malaria, increased ICP has been observed in a substantial proportion of cases, with estimates ranging from 10% to 15% demonstrating papilledema, indicating optic disc swelling caused by elevated ICP [3]. A small study of 23 Kenyan children with CM found that 100% had raised ICP at the time of diagnosis [3]. A portion of the children in this study developed severe intracranial hypertension, all of whom subsequently died or had neurological sequelae [4]. The presence of increased ICP in cerebral malaria necessitates prompt and aggressive management. However, the challenges in resource-limited settings, such as the lack of advanced neuroimaging, limit the ability to assess and monitor ICP. Consequently, alternative methods, such as point-of-care ultrasound (POCUS), have been explored for their potential in evaluating optic disc changes as a surrogate marker for increased ICP. 

In this case report, we present a case of a 14-year-old boy with cerebral malaria who exhibited optic disc elevation, indicating elevated ICP. Due to the unavailability of advanced neuroimaging in the rural Liberian setting, POCUS was employed to assess the optic disc and monitor treatment response. The resolution of optic disc elevation following mannitol administration correlated with clinical improvement in our patient, demonstrating the utility of POCUS as a feasible tool for assessing and managing cerebral malaria in resource-limited settings.

## Case presentation

**Figure 1:**
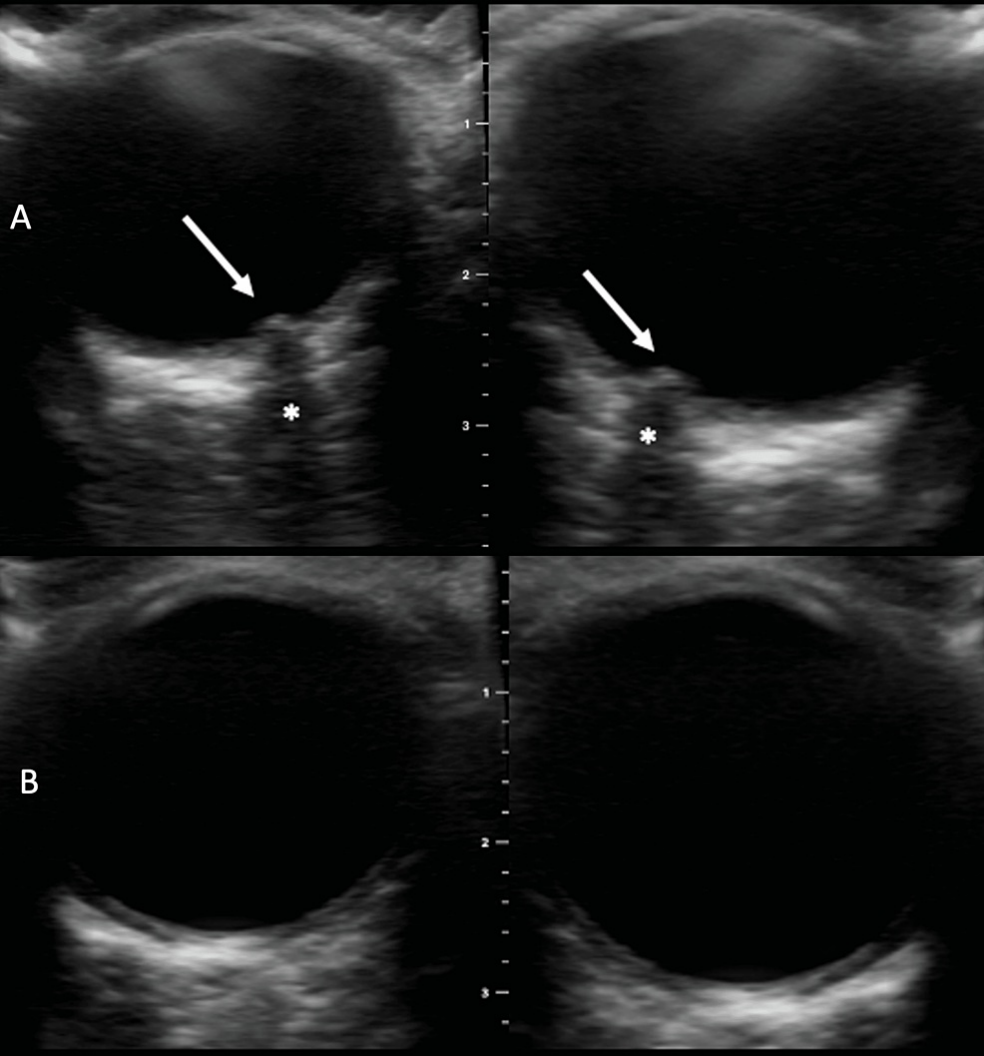
B-scan ultrasound image. 1A demonstrates marked optic disc elevation in the globes indicating raised intracranial pressure (arrow). Optic nerves indicated by *. 1B demonstrates resolution of optic disc elevation following the administration of mannitol for the management of increased intracranial pressure.

An otherwise healthy 14-year-old boy presented to the emergency department in rural Liberia febrile, tachycardic, and comatose. Family members reported a 48-hour history of tactile fever at home, several episodes of non-bloody emesis, and two convulsions en route to the hospital. At triage his vital signs were as follows: heart rate of 131 beats per minutes, respiratory rate of 24, temperature of 38.3 degrees Celcius and blood pressure of 145/73 mmHg. On physical examination, his eyes were closed, he localized to noxious stimuli, and had no verbal response. There were no obvious signs of meningismus on physical examination. The rest of the patient’s physical examination was unremarkable. The point of care glucose was 89 mg/dL. Initial laboratory investigations were ordered which included a malaria rapid diagnostic test which was positive. The patient was treated empirically with broad-spectrum antibiotics to cover for meningitis and IV artesunate for cerebral malaria. Peripheral intravenous access was established and the patient was given a weight-based bolus of crystalloid. Given the patient’s clinical presentation there was concern for increased intracranial pressure (ICP) causing his decreased level of consciousness. Unfortunately, there is no availability of advanced neuroimaging in this area of Liberia or the requisite tools to perform lumbar puncture. POCUS was performed which showed marked bilateral optic disc elevation potentially indicating raised intracranial pressure (Figure 1A) [5]. A weight-based dose of mannitol was administered and 30 minutes later the POCUS was repeated (Figure 1B). On repeat examination optic disk elevation had resolved completely and shortly thereafter the patient began to respond to verbal stimuli. The patient was subsequently transferred to the pediatrics ward.

## Discussion

Lumbar puncture is often the preferred diagnostic and therapeutic procedure used in the context of assessing and managing ICP [6]. However, there are potential complications associated with lumbar puncture, particularly in resource-limited settings. These complications include the risk of infection, bleeding, and neurological injury. Performing a lumbar puncture in critically ill patients with cerebral malaria can pose additional challenges due to the risk of worsening neurological status, difficulties in patient positioning, and limited availability of equipment for the procedure. However, POCUS emerged as a valuable alternative for monitoring optic disc changes. 

Ocular POCUS has demonstrated utility and has been validated for the measurement of optic nerve sheath diameter (ONSD) in adults, allowing for the assessment of ICP [7]. However, in pediatric populations no consensus exists on normal pediatric ONSD values. Nonetheless, optic disc elevation (ODE) has been validated as a reliable indicator of increased ICP in pediatric patients from ages 0 to 18 years [5]. Cerebral malaria commonly causes increased ICP although the underlying pathophysiology is not well understood [8]. There is however a strong correlation between increased ICP and mortality in pediatric cerebral malaria and therefore aggressive management of ICP is warranted [8]. As well, the strong association between cerebral malaria (and coma secondary to such) with malarial retinopathy has been well demonstrated, with duration of coma being correlated with the severity of retinopathy [9]. To our knowledge this is the first reported case of serial point-of-care ultrasound imaging of optic disc elevation resolution in presumed cerebral malaria. The use of POCUS allowed for non-invasive monitoring of optic disk changes as a surrogate for ICP, enabling the assessment of treatment response and clinical improvement. 

Serial POCUS monitoring of optic disc elevation offers several advantages in the management of cerebral malaria. Firstly, it enables real-time evaluation of changes in ICP, allowing for timely adjustments in therapeutic interventions. Secondly, it provides a non-invasive and safe method of monitoring without exposing the patient to the risks associated with lumbar puncture. This is especially crucial in critically ill pediatric patients with cerebral malaria, where minimizing additional risks is of paramount importance.

It is also important to acknowledge the limitations of POCUS in the context of assessing ICP. While optic disc elevation serves as an indirect marker for increased ICP, it does not provide direct measurement or detailed information about the absolute ICP values. Further research to establish standardized pediatric ONSD values and validate the use of POCUS as a reliable tool for monitoring ICP is warranted. Future investigations should aim to elucidate the correlation between optic disc changes observed through POCUS and actual ICP measurement. 

## Conclusions

This case highlights the utility of POCUS as a feasible and safe alternative for serial monitoring of ODE in pediatric cerebral malaria and underscores potential complications associated with lumbar puncture in resource-limited settings. POCUS provides valuable information for assessing treatment response and clinical improvement while avoiding the risks associated with invasive procedures. In this case, optic disc elevation resolution preceded clinical improvement in the management of a 14-year-old boy with severe malaria. 
